# Side effect of acting on the world: acquisition of action-outcome statistic relation alters visual interpretation of action outcome

**DOI:** 10.3389/fnhum.2013.00610

**Published:** 2013-09-25

**Authors:** Takahiro Kawabe

**Affiliations:** NTT Communication Science LaboratoriesAtsugi, Japan

**Keywords:** action, causality, prediction, visual motion, sense of agency

## Abstract

Humans can acquire the statistical features of the external world and employ them to control behaviors. Some external events occur in harmony with an agent's action, and thus, humans should also be able to acquire the statistical features between an action and its external outcome. We report that the acquired action-outcome statistical features alter the visual appearance of the action outcome. Pressing either of two assigned keys triggered visual motion whose direction was statistically biased either upward or downward, and observers judged the stimulus motion direction. Points of subjective equality (PSE) for judging motion direction were shifted repulsively from the mean of the distribution associated with each key. Our Bayesian model accounted for the PSE shifts, indicating the optimal acquisition of the action-effect statistical relation. The PSE shifts were moderately attenuated when the action-outcome contingency was reduced. The Bayesian model again accounted for the attenuated PSE shifts. On the other hand, when the action-outcome contiguity was reduced, the PSE shifts were greatly attenuated, and however, the Bayesian model could not accounted for the shifts. The results indicate that visual appearance can be modified by prediction based on the optimal acquisition of action-effect causal relation.

## Introduction

Humans can acquire statistical features of external events and use them to accommodate their behaviors. For example, statistical features in temporal (Miyazaki et al., 2006a; Acerbi et al., 2012) and spatial (Tassinari et al., 2006; Vilares et al., 2012) sensory stimuli can be acquired, and the acquired statistical features significantly alter manual responses in sensorimotor tasks. Moreover, acquiring statistical features for the temporal aspect of sensory signals can also affect temporal order judgments for the signals (Miyazaki et al., 2006b; Nagai et al., 2012; Yamamoto et al., 2012). These results suggest that the brain can use the acquired statistical features as prior knowledge about the external world to choose and execute appropriate responses to the world.

Such prior knowledge about the world can also alter visual perception (Freeman, 1994). For example, implicit prior knowledge about the position of a light source can affect the perception of three-dimensional surface shapes (Mamassian and Goutcher, 2001; Adams et al., 2004; Gerardin et al., 2010) [see Kersten et al. (2006) for a review]. Prior knowledge that affects the perception of the world can also be optimally learned (Orban et al., 2006).

Some visual events are caused by an agent's action. For example, we see a line being drawn on paper with the stroke of a pen. We obviously have prior knowledge about the relation between the action (i.e., drawing) and its outcome (a drawn line). So far, researchers (Körding and Wolpert, 2004) have focused on how the prior knowledge between an action and outcome could accommodate manual responses in a sensory motor task. On the other hand, another important question, which has not been addressed, is whether the acquisition of statistical relationships between an action and its outcome influence the interpretation of action outcome. In addition, it was also an open question whether such acquisition of action-effect statistical relationships was statistically optimal. In this work, three experiments were conducted to resolve these issues.

## Experiment 1

### Background

**Figure 1:**
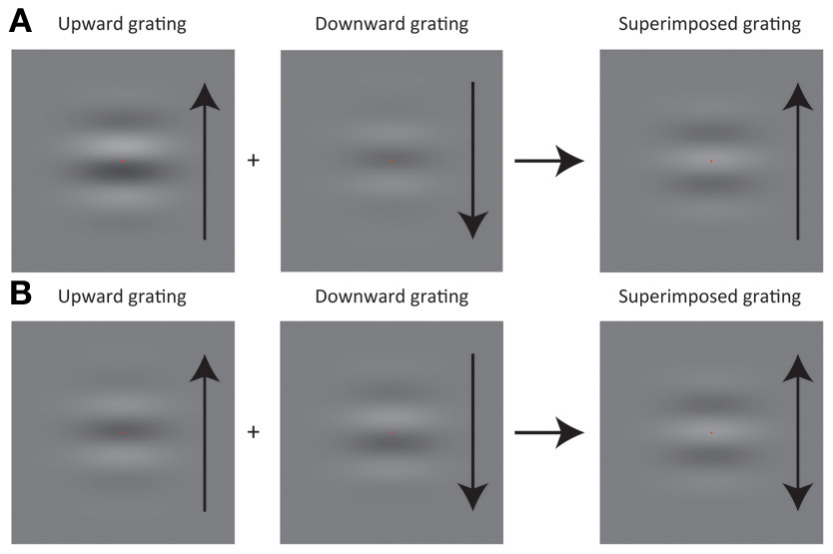
**Schematic illustrations of grating stimuli used in experiments. (A)** When a high contrast upward (downward) grating is superimposed on a low-contrast downward (upward) grating, a percept of an upward (downward) grating results in. **(B)** If the contrast of an upward grating is equivalent to the one of a downward grating, a grating with ambiguous motion direction is perceived.

**Figure 2:**
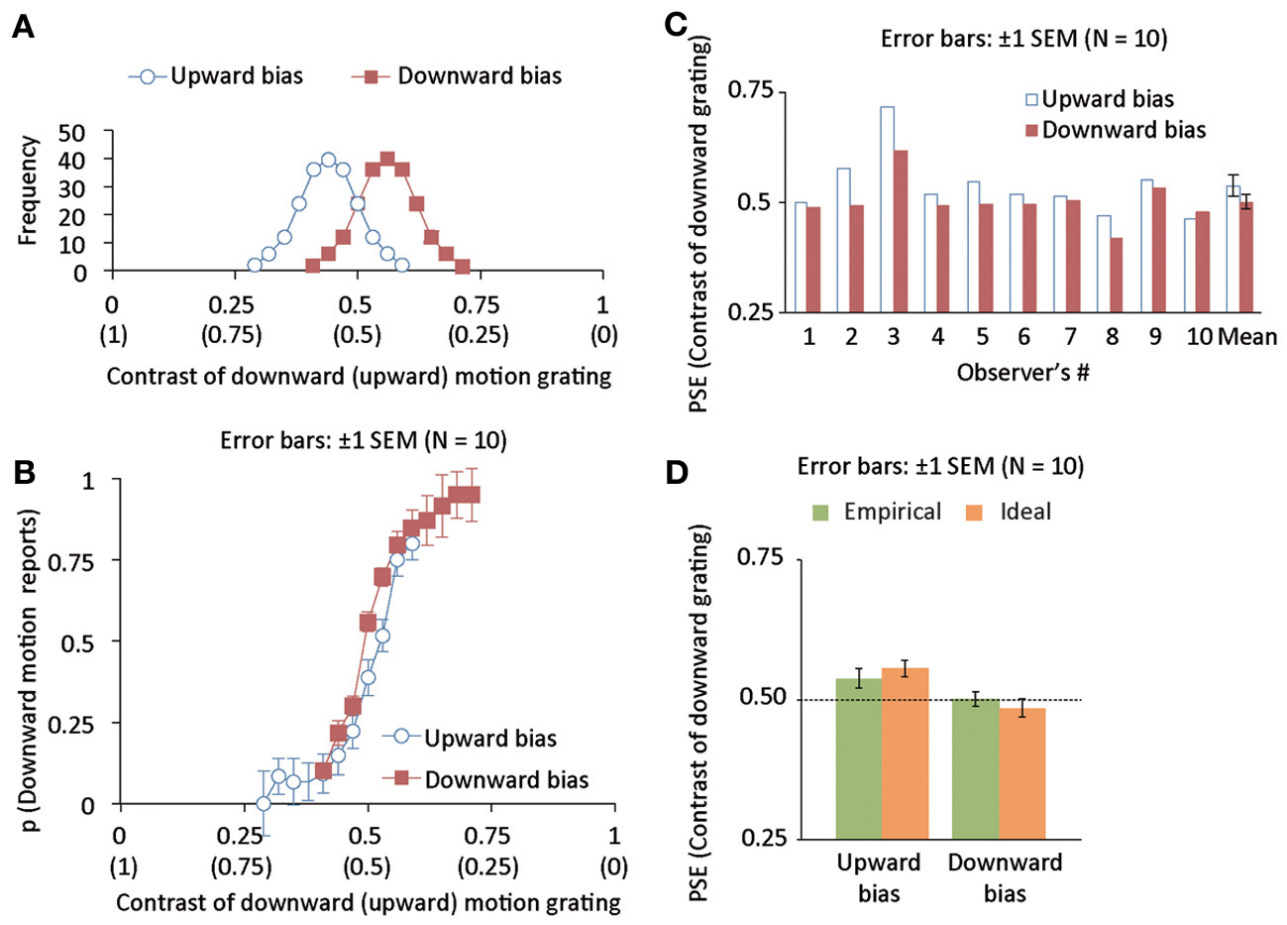
**(A)** Distributions of luminance contrast for downward (and upward) drifting gratings in upward (blue, open disk maskers) and downward (red, open square markers) bias conditions. The mean of the distribution is deviated from 0.5 by 0.06 negatively and positively for upward- and downward-bias conditions. The standard deviation of the distribution was 0.06. **(B)** The proportion of trials wherein drifting direction was reported to be downward as a function of the luminance contrast of a downward grating in Experiment 1. **(C)** Individual and group data of empirical PSEs in Experiment 1. **(D)** Mean ideal and empirical PSEs in Experiment 1.

The purpose of this experiment was to explore whether the acquisition of the statistical relation between an action and its outcome would distort the interpretation of the action outcome. Observers were asked to press assigned keys to trigger a drifting grating as an action outcome on a CRT display. The task of the observers was to report whether motion direction was upward or downward. As depicted in Figure 1, we spatially superimposed upward and downward drifting gratings and manipulated their luminance contrast (e.g., when the contrast of an upward grating was ω, the contrast for a downward grating was 1-ω). It was expected that judged motion direction in the superimposed grating would be consistent with the motion direction of the component drifting grating having stronger luminance contrast (Figure 1A, see also Movies 1 and 2). We also expected that a superimposed grating, where each component grating has the contrast of 0.5 would likely result in an ambiguous judgment of motion direction (Figure 1B, see also Movie 3). In the experiment, the luminance contrast in the superimposed drifting grating was dependent on which keys the observers pressed. For example, when the observers pressed left and right keys (though a reverse key mapping was also tested), the relative contrast of each component grating was chosen from a Gaussian distribution (i.e., prior distribution) where its mean was biased so that the downward grating on average had lower and higher relative contrast than the upward grating (Figure 2A). If the observers could really learn the statistical relation between key press and visual motion direction, the point of subjective equality (PSE) for motion direction would be biased repulsively from the mean of the prior distribution that was associated with either key. Moreover, employing a computational model based on Bayesian statistics, we tested whether the acquisition of action-effect statistical relation was statistically optimal.

### Methods

#### Observers

Ten naive people (6 females and 4 males) served as observers. They reported they had normal or corrected-to-normal visual acuity. They were paid 1000 JPY for their participation. Ethical approval for this study was obtained from the ethical committee at Nippon Telegraph and Telephone Corporation (NTT Communication Science Laboratories Ethical Committee). The experiments were conducted according to the principles laid down in the Helsinki Declaration. Written informed consent was obtained from all participants in this study.

#### Apparatus

Stimuli were presented on a 21-inch CRT monitor (GDM-F500R, Sony) with the resolution of 1024 × 768 pixels (38 × 30 cm) and refresh rate of 100 Hz. A photometer (OP200-E, Cambridge Research Systems) linearized the luminance emitted from the monitor in a range from 0 to 106 cd/m^2^. A computer (Mac Pro, Apple) controlled stimulus generation, stimulus presentation, and data collection. Stimuli were generated by using MATLAB and PsychToolBox 3 (Brainard, 1997; Pelli, 1997).

#### Stimuli

We used horizontally oriented sinusoidal drifting gratings as stimuli (Figure 1). Each grating was windowed by a two-dimensional Gaussian function with the standard deviation of 3.58 degrees of visual angle. The spatial frequency of the gratings was 0.22 cycles per degree. Each of eight frames of drifting gratings lasted for 100 msec. Because no temporal interval was inserted between successive frames, the whole drifting-grating presentation lasted for 800 msec. The phase of the grating was shifted upward/downward by 0.5π per frame, and thus, drifting frequency was 2.5 Hz. The drifting speed was 11.2°/s. In the upward-bias condition, the contrast of a downward grating was chosen from the following alternatives: 0.29, 0.32, 0.35, 0.38, 0.41, 0.44, 0.47, 0.50, 0.53, 0.56, and 0.59, which were presented 2, 6, 12, 24, 36, 40, 36, 24, 12, 6, and 2 out of 200 trials, respectively, (see Figure 2A for the contrast relationship between the upward- and downward-bias conditions). The frequency of trials as a function of the contrast of a downward grating followed a Gaussian distribution with a mean of -0.06 and a standard deviation of 0.06. In the downward-bias condition, the contrast of a downward grating was chosen from the following alternatives: 0.41, 0.44, 0.47, 0.50, 0.53, 0.56, 0.59, 0.62, 0.65, 0.68, 0.71, which were presented 2, 6, 12, 24, 36, 40, 36, 24, 12, 6, and 2 out of 200 trials, respectively (Figure 2A). The frequency of trials as a function of the contrast of a downward grating followed a Gaussian distribution with a mean of 0.06 and a standard deviation of 0.06. In each condition, values after subtracting the contrast of the downward grating from 1 were given as the luminance contrast of an upward grating. The downward grating was superimposed on the upward grating. Consequently, the superimposed grating was presented to the observer as a stimulus.

#### Procedure

Participants sat 70 cm from the CRT display. In each trial, they were asked to press one of two keys (“Z” and “M”) with the index finger of the left and right hands, respectively. They were allowed to freely choose the key to press on their own. Pressing the key triggered the drifting grating in the display. For half of the observers, left and right keys produced the drifting grating with the relative contrast chosen from alternatives in the upward- and downward-bias conditions, respectively, and the reverse was true for the other half. The observers were asked to pay attention to the drifting grating, and after the disappearance of a drifting grating, to judge direction in which (upward or downward) the drifting grating moved. They pressed “T” and “V” keys when they saw upward and downward motion, respectively. No feedback was given to the observers. Digits were provided at the left and right bottom of the display to help the observers notice the number of trials in which they pressed “Z” and “M” keys. It took 30–40 min for each observer to complete an experimental session, which consisted of 400 trials. The order of trials was randomized.

### Results and discussion

We calculated the proportion of trials in which downward motion was perceived as a function of the relative contrast of the downward-drifting grating, and averaged the proportion across observers (Figure 2B). We individually fitted a cumulative Gaussian function to the proportion data and computed the relative contrast causing 50% responses of downward motion as an empirical PSE for motion direction (Empirical PSE in Figure 2C). Consequently, the PSE was significantly different between upward- and downward-bias conditions [*t*_(9)_ = 3.22, *p* < 0.011, Cohen's *d* = 0.57]. Next, we tried to assess the difference between empirical and ideal PSEs. In a way similar to previous studies (Miyazaki et al., 2006a,b; Nagai et al., 2012; Yamamoto et al., 2012), we used a Bayesian model (see Appendix for the detail of the model) to estimate the ideal PSEs on the basis of the Bayesian statistics. Using the empirical and ideal PSEs as plotted in Figure 2D, we conducted a mixed two-way repeated measures ANOVA with the data source (model and empirical observers) as a between-subject factor and bias direction (upward and downward) as a within-subject factor. The main effect of the data source was not significant [*F*_(1, 18)_ = 0.000, *p* = 0.98]. On the other hand, the main effect of bias direction was highly significant [*F*_(1, 9)_ = 55.131, *p* < 0.0001]. Interaction between the two factors was also significant [*F*_(1, 18)_ = 5.877, *p* < 0.03]. Simple main effect of the data source was still not significant in the upward [*F*_(1, 18)_ = 0.420, *p* > 0.05] and downward [*F*_(1, 18)_ = 0.378, *p* > 0.05] bias conditions. Simple main effect of the bias direction was significant in the empirical [*F*_(1, 18)_ = 12.504, *p* < 0.03] and ideal [*F*_(1, 18)_ = 48.505, *p* < 0.03] observers. The results suggest that the human brain can acquire the statistical relation between an action and its outcome in a statistically optimal manner, and consequently alter the judgment for the appearance of the action outcome.

## Experiment 2

### Background

**Figure 3:**
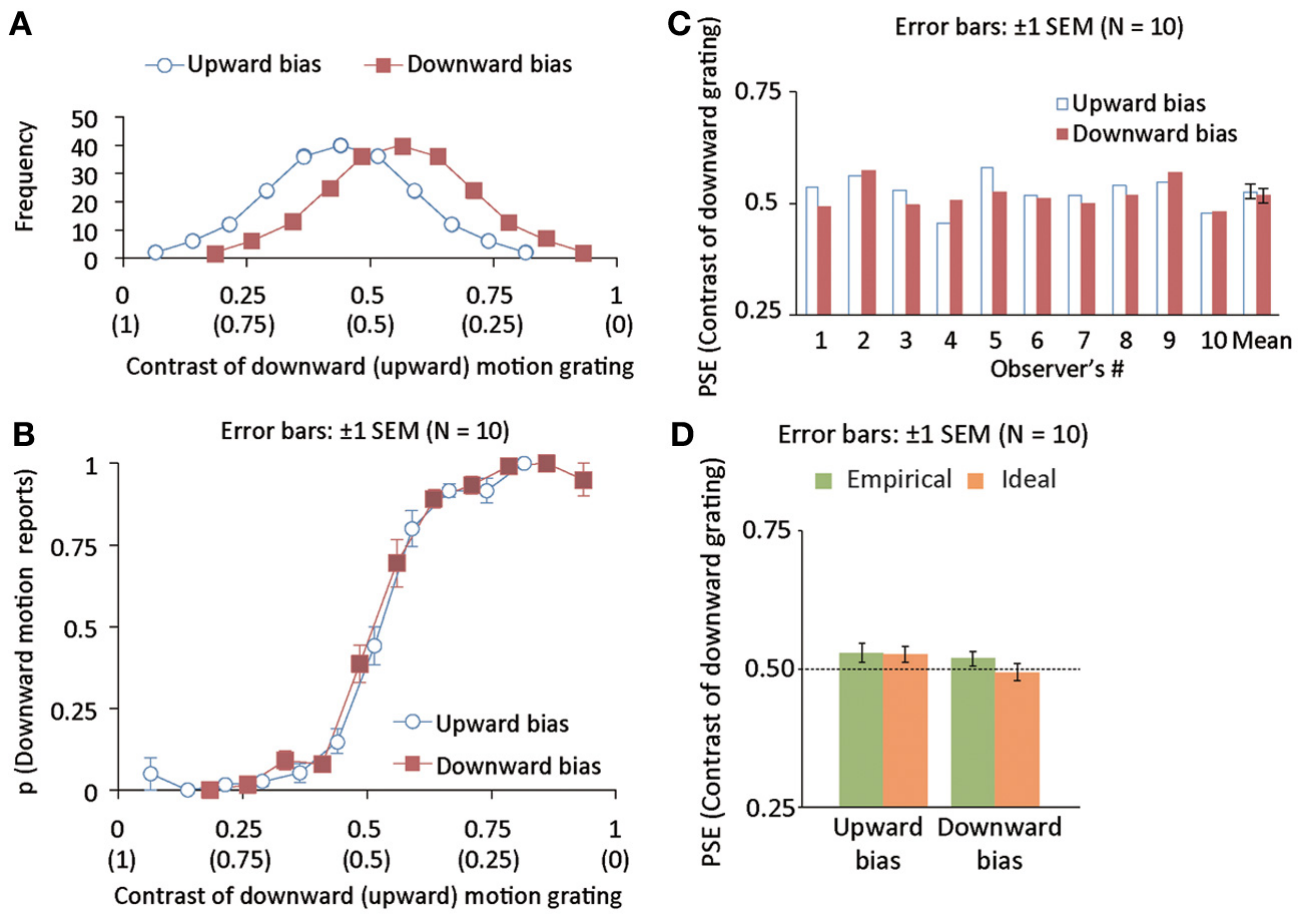
**(A)** Distributions of luminance contrast for downward (and upward) drifting gratings in upward (blue, open disk maskers) and downward (red, open square markers) bias conditions. The mean of the distribution is deviated from 0.5 by 0.06 negatively and positively for upward- and downward-bias conditions. The standard deviation of the distribution was 0.15. **(B)** The proportion of trials wherein drifting direction was reported to be downward as a function of the luminance contrast of a downward grating in Experiment 2. **(C)** Individual and group data of empirical PSEs in Experiment 2. **(D)** Mean ideal and empirical PSEs in Experiment 2.

The acquisition of an action-outcome relation will be strongly attenuated when the prior distribution (i.e., the Gaussian distribution of a relative contrast in a superimposed grating) is wide, consistent with a previous study (Miyazaki et al., 2006a). To confirm this prediction, using a new group of 10 observers (5 females and 5 males), we tested whether the PSE shift as observed in experiment 1 is reduced when the standard deviation of the prior distribution is increased from 0.06 to 0.15. (compare Figure 2A with Figure 3A). Except for the standard deviation manipulation, the stimuli and procedure were identical to those in experiment 1.

### Results and discussion

We calculated the proportion of trials in which downward motion was perceived as a function of the contrast of the grating with a downward motion (Figure 3B), and calculated the empirical PSE as we did in experiment 1 (Figure 3C). The PSE was not significantly different between the two bias conditions [*t*_(9)_ = 0.92, *p* = 0.38]. To check the difference in the PSE between Experiments 1 and 2, we conducted a two-way mixed repeated measures analysis of variance (ANOVA) with distribution width as a between-subject factor and bias direction as a within-subject factor. The main effect of the distribution width was not significant [*F*_(1, 18)_ = 0.018, *p* = 0.89]. The main effect of the bias direction was significant [*F*_(1, 18)_ = 9.338, *p* < 0.007]. Interaction between the two factors was marginally significant [*F*_(1, 18)_ = 3.083, *p* < 0.097]. Based on the outcome of *t*-test and ANOVA, we suggest that the PSE shifts based on the acquisition of action-effect relations are moderated with a larger width of the prior distribution. To check whether the Bayesian model could account for the attenuation of the PSE shifts, we assessed the statistical difference between ideal and empirical PSEs. Using the empirical and ideal PSEs as plotted in Figure 3D, we conducted a mixed two-way repeated measures ANOVA with the data source (model and empirical observers) as a between-subject factor and bias direction (upward and downward) as a within-subject factor. The main effect of the data source was not significant [*F*_(1, 18)_ = 0.886, *p* = 0.3590]. On the other hand, the main effect of bias direction was highly significant [*F*_(1, 9)_ = 12.193, *p* < 0.0026]. Interaction between the two factors was significant only marginally [*F*_(1, 18)_ = 3.403, *p* < 0.082]. The acquisition of the action-effect relation was not removed but attenuated with the large standard deviation of the prior distribution while our Bayesian model could account for the magnitude of the attenuation. Taken together, the results again indicate the optimal acquisition of the action-effect statistical relation.

## Experiment 3

### Background

**Figure 4:**
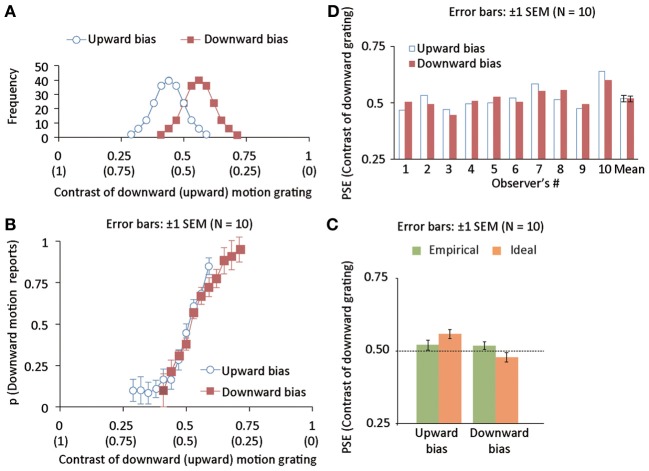
**(A)** Distributions of luminance contrast for downward (and upward) drifting gratings in upward (blue, open disk maskers) and downward (red, open square markers) bias conditions. The mean of the distribution is deviated from 0.5 by 0.06 negatively and positively for upward- and downward-bias conditions. The standard deviation of the distribution was 0.06. **(B)** The proportion of trials wherein drifting direction was reported to be downward as a function of the luminance contrast of a downward grating in Experiment 3. **(C)** Individual and group data of empirical PSEs in Experiment 3. **(D)** Mean ideal and empirical PSEs in Experiment 3.

An external event is recognized as the outcome of one's own action when a temporal discrepancy between the action and the event is small (Berberian et al., 2012; Kawabe et al., 2013). An association between an action and its outcome is also established depending strongly on the temporal contiguity between them (Elsner and Hommel, 2004). Moreover, it is known that one critical determinant of associative learning is the temporal contiguity between the response and outcome (Wasserman and Miller, 1997). On the basis of these lines of evidence, we predicted that inserting a delay between an action and outcome might hamper the acquisition of an action-outcome statistical relation even when the prior distribution is sufficiently narrow because the delayed event following an agent's action is possibly no longer an action outcome for the brain (Berberian et al., 2012; Kawabe et al., 2013). Using a completely new group of 10 observers (6 females and 4 males), we examined whether human observers can acquire an action-effect statistical relation (Figure 4A) even when a 2-s delay is inserted between action and outcome.

### Results and discussion

We calculated the proportion of trials in which downward motion was perceived as a function of the contrast of the grating with a downward motion (Figure 4B) and calculated the empirical PSE as we did in experiment 1 (Figure 4C). As a result, we found that the PSE was not significantly different between the two bias conditions [*t*_(9)_ = 0.22, *p* = 0.83]. To check the difference in the PSE between Experiments 1 and 3, we conducted a two-way mixed repeated measures analysis of variance (ANOVA) with action-effect delay (i.e., the delay was absent in Experiment 1 while was present in Experiment 3) as a between-subject factor and bias direction as a within-subject factor. The main effect of the presence/absence of the action-effect delay was not significant [*F*_(1, 18)_ = 0.002, *p* = 0.96]. The main effect of the bias direction was significant [*F*_(1, 18)_ = 6.647, *p* < 0.019]. Interaction between the two factors was significant [*F*_(1, 18)_ = 5.117, *p* < 0.04]. Simple main effect of the bias condition was significant only when there was no delay between action and outcome (i.e., in Experiment 1) [*F*_(1, 18)_ = 11.421, *p* < 0.004], but not when there was an action-outcome delay (i.e., in Experiment 3) [*F*_(1, 18)_ = 0.050, *p* = 0.82]. These results indicate that inserting a delay between action and outcome causes the significant attenuation in the acquisition of action-effect statistical relation. To see the relation between ideal and empirical PSEs, we assessed the statistical difference between them. Using the empirical and ideal PSEs as plotted in Figure 4D, we conducted a mixed two-way repeated measures ANOVA with the data source (model and empirical observers) as a between-subject factor and bias direction (upward and downward) as a within-subject factor. The main effect of the data source was not significant [*F*_(1, 18)_ = 0.001, *p* = 0.9711]. On the other hand, the main effect of bias direction was highly significant [*F*_(1, 9)_ = 52.314, *p* < 0.0000]. Interaction between the two factors was highly significant [*F*_(1, 18)_ = 46.369, *p* < 0.0001]. Simple main effect of the bias direction was significant for the ideal PSEs [*F*_(1, 18)_ = 98.593, *p* < 0.0001], but not for the empirical PSEs [*F*_(1, 18)_ = 0.090, *p* < 0.07681]. The Bayesian model predicted the significant difference in the PSEs between two bias conditions while empirical data demonstrated that the PSEs were not different between the two conditions. To sum up, these results indicate the following two points; first, acquiring an action-outcome relation is strongly reduced when a large delay is inserted between an action and its outcome, and second, the large delay between action and outcome hinders the optimal acquisition of action- outcome statistical relationship. It has been suggested that a 2-s delay is sufficient to greatly reduce the sense of agency (or sense of causality) for external events (Berberian et al., 2012; Kawabe et al., 2013). Because an agent does not likely consider the event (i.e., drifting grating) as a causal outcome of her/his action when delay is inserted between an action and its outcome, only a weak acquisition of an action-outcome relation possibly results in.

## General discussion

Consistent with previous studies (Körding and Wolpert, 2004), we observed that the human observers can optimally acquire the action-effect relationship. On the other hand, we recently found that the acquisition of an action-effect relation has a side effect: visual interpretation of action outcome is strongly modulated by the acquired relation between an action and its outcome. However, the acquisition effect on the interpretation of action outcome was moderately attenuated when the width of the distribution to be acquired was large, and moreover, was greatly attenuated when there was a temporal delay between the action and its effect. These results indicate that the acquisition of a statistical relation between an action and its outcome clearly depends on the consistency (experiment 2) and contiguity (experiment 3) between action and its effect.

It is already known that acquiring the statistical relation between visual events strongly alters the perception of motion direction (Gekas et al., 2013). Moreover, it has been shown that motion direction perception is strongly affected by an action-effect relation that is naturally acquired through one's development (Wohlschläger, 2000; Maruya et al., 2007). Beyond these studies, the present study suggests that such modulation of visual motion perception by action occurs as a result of motion prediction from the acquired statistical relation between an action and its outcome. A previous study (Jordan and Hunsinger, 2008) has reported that the learned pattern of action outcome can enhance the forward mislocalization of a moving target, but it did not address the statistical aspects of the action-outcome relation. We suggest that the successful acquisition of an action-outcome's statistical relationship can trigger the prediction for visual motion direction that is associated with action, and consequently alter the appearance of visual motion, while it is still unclear whether perceptual bias or response bias is triggered by the action-related prediction of visual motion. Anyway, we speculate that spontaneous cortical activities, which are promising neural correlates of prior representation (Berkes et al., 2011; De Lange et al., 2013), possibly mediate the expectation for motion direction on the basis of an action-outcome relationship.

An intriguing future issue is whether an endogenous action is a necessary factor for acquiring the action-outcome statistical relation. In learning the relation between action and its outcome, endogenous and exogenous actions respectively, contribute to ideomotor and sensorimotor learnings (Herwig et al., 2007; Herwig and Waszak, 2012). In particular, endogenous action seems to trigger a long-term association between an action and its outcome. In this respect, an endogenous action may be an important factor for efficiently learning the action-effect statistical relation. On the other hand, another line of research has demonstrated that human observers can learn the statistical relationship between spatial cues and a tactile temporal order judgment without executing any action (Nagai et al., 2012), suggesting that the statistical relation between external events can be acquired if subjective causality is established between two events. In the present study, we found that the acquisition of action-outcome statistical relation deteriorates when the congruency and temporal contiguity between action and outcome, which presumably play a fundamental role in causality perception, are reduced (Hume, 1888; Wegner, 2005; Woods et al., 2012; Kawabe, 2013; Kawabe et al., 2013). Thus, it is also possible that the perception of causality between an action and its outcome is one of the decisive factors for the acquisition of their statistical relation. Other lines of evidence have suggested that causality inference between events plays critical roles in the optimal integration of cross-sensory signals (Körding et al., 2007; Sato et al., 2007; Berniker and Körding, 2011). As such, we suggest that the perception of causality between an action and its outcome at least partly underlies the acquisition of the statistical relation between them, though we need to empirically dissociate the contribution of action from non-action factors to the acquisition of an action-effect relation.

## Supplementary material

The Supplementary Material for this article can be found online at: http://www.frontiersin.org/Human_Neuroscience/10.3389/fnhum.2013.00610/abstract

### Conflict of interest statement

The author declares that the research was conducted in the absence of any commercial or financial relationships that could be construed as a potential conflict of interest.

## Appendix

We checked whether the acquisition of the action-outcome relation was statistically optimal. In a way similar to previous studies (Miyazaki et al., 2006a,b; Nagai et al., 2012; Yamamoto et al., 2012), we used a Bayesian model, where the probability of possible true motion direction *d*_true_ given the sensed motion direction *d*_sensed_ is expressed as

(1) $$p\left(d_{\text{true}}|d_{\text{sensed}}\right)=p_{\text{prior}}\left(d_{\text{true}}\right)p\left(d_{\text{sensed}}|d_{\text{true}}\right)/p\left(d_{\text{sensed}}\right)$$

where *d*_sensed_ denotes the sensory measurement of the true motion direction that distributes in a Gaussian manner with a mean of zero and a standard deviation of σ_sensed_, and the *p*_prior_ (*d*_true_) denotes the prior distribution for motion direction, which follows a Gaussian distribution expressed as

(2) $$p_{\text{prior}}\left(d_{\text{true}}\right)=\text{G}\left(d_{\text{true}};\mu_{\text{prior}},\sigma_{\text{prior}}\right)$$

where μ_prior_ = ±0.06 in terms of the contrast of a downward drifting grating, and σ_prior_ = 0.06 in experiments 1 and 3 and 0.15 in experiment 2. Finally, the motion direction is optimally estimated so as to maximize the left side term *p*(*d*_true_|*d*_sensed_) in (1). Here, optimal judgment on motion direction is based on the weighted sum of μ_prior_ and d_sensed_, which is expressed as

(3) $$d_{\text{judged}}=(1-\omega )\mu_{\text{prior}}+\omega d_{\text{sensed}}$$

where

(4) $$\omega =\sigma_{\text{prior}}^{2}/\left(\sigma_{\text{prior}}^{2}+\sigma_{\text{sensed}}^{2}\right)$$

Moreover, a perceptual bias in vertical motion direction has been reported, which likely occurs independently from the weighted sum described above (Gros et al., 1998; Naito et al., 2010). We believed we should consider this factor of perceptual bias in our model. The perceptual bias β was calculated by averaging the deviations of PSEs from 0.5 in both upward and downward direction conditions. Thus, the final estimate of perceived motion direction is expressed as

(5) $$d_{\text{judged}}=(1-\omega )\mu_{\text{prior}}+\omega d_{\text{sensed}}+\beta$$

β s in Experiments 1, 2, and 3 were 0.020 (SEM: 0.019), 0.02 (SEM: 0.009), and 0.018 (SEM: 0.015), respectively. The positive β s indicate a consistent bias toward upward motion reports.
